# Bridging Molecular Modeling Insights and Experimental Findings: A Comparative Study on Surfactant Effects in Al_2_O_3_ Nanofluids

**DOI:** 10.3390/nano16020092

**Published:** 2026-01-11

**Authors:** Beytullah Erdoğan, Çağlar Çelik Bayar

**Affiliations:** 1 Department of Mechanical Engineering, Zonguldak Bülent Ecevit University, 67100 Zonguldak, Türkiye; 2 Department of Metallurgical and Materials Engineering, Zonguldak Bülent Ecevit University, 67100 Zonguldak, Türkiye; caglarbayar@beun.edu.tr

**Keywords:** Al_2_O_3_ nanofluid, surfactant, zeta potential, thermal conductivity, DFT, LANL2DZ

## Abstract

This study aimed to prepare water-based nanofluids using Al_2_O_3_ nanoparticles with different types of surfactants, and to investigate the colloidal and thermophysical properties of the obtained nanofluids. In this context, water-based Al_2_O_3_ nanofluids have been prepared using six surfactants with anionic, cationic, and nonionic characteristics SDS, CTAC, PVP, Tween 80, PVA, and Triton X-100. The electrostatic colloidal stability of the prepared samples has been determined by zeta potential and particle size measurements. To understand the interactions at the molecular level and the stabilities in terms of interaction Gibbs free energy, nanoparticle–surfactant interactions have been modeled using the DFT (Density Functional Theory) method. The overall colloidal stability rankings of nanofluids have been performed using both zeta potential measurements and DFT analysis. Furthermore, the thermophysical properties of nanofluids, which are crucial for industrial applications, have been measured. The results showed that the type of surfactant has a significant effect on the colloidal and thermophysical properties of nanofluids. It has been concluded that Al_2_O_3_-SDS and Al_2_O_3_-CTAC nanofluids can be used in cooling systems due to their high zeta potential and thermal conductivity, and low viscosity and size.

## 1. Introduction

Water-based nanofluids (Al_2_O_3_, TiO_2_, ZnO, CuO, etc.) have become an important research topic in heat transfer applications in recent years [1,2,3]. However, the tendency of nanoparticles to precipitate over time in heating or cooling systems limits both the reliability of the measurement of their thermophysical properties and practical use. Therefore, the use of surfactants has emerged as a common approach to increase stability. Recent studies in the literature reveal that surfactants retard precipitation by increasing the zeta potential value [4]. Particularly, anionic SDS and cationic CTAB surfactants are effective in improving stability. It has been reported that polymeric surfactants (such as polymer-based PVP) provide longer-term stability but can negatively affect the rheological properties of the fluid [5]. Furthermore, it has been stated that determining the surfactant concentration ratio is important. Low concentration ratios increase the stability of the fluid, and in contrast, high concentration ratios can limit heat transfer between nanoparticles [6,7].

When nanofluids are evaluated in terms of thermal conductivity, it is observed that the use of surfactants yields varying results. While some experimental studies have shown a modest increase in thermal conductivity with the addition of surfactants to nanofluids [8], other studies have reported higher thermal conductivity values in nanofluids without surfactants [9,10]. It is emphasized that this is due to variables such as the type and concentration of the surfactant used, nanoparticle size and surface properties, and nanofluid preparation methods [11]. Especially in hybrid systems (e.g., Al_2_O_3_-TiO_2_), surfactants play an important role not only in thermal conductivity but also in interactions between nanoparticles [12]. The results show that surfactants improve the stability of nanofluids, but their effects on thermal conductivity do not always provide a systematic increase.

In terms of dynamic viscosity, surfactants have generally been reported to have an enhancing effect. While ionic surfactants exhibit a lower increase in dynamic viscosity at lower concentrations, polymeric surfactants exhibit a higher increase in dynamic viscosity [13]. The need for pumping power may rise as a result of the increase in dynamic viscosity [14]. To balance increased heat transfer and increased pumping power, some research has demonstrated that the viscosity increase can be managed by choosing the right surfactant and concentration ratio. These conflicting findings indicate that the type and amount of surfactant play a critical role not only in stability but also in thermophysical properties. Therefore, it is revealed that there are still some vacancies in the literature that require research on the determination of optimum surfactant selection and concentration in Al_2_O_3_-water nanofluids [15,16,17].

The electrostatic charge that forms on the surface of tiny particles in a nanofluid is measured by zeta potential, which provides information about the stability of the nanofluid. Particles tend to repel each other and clump together less when the zeta potential reaches higher values. As a result, the nanofluid can maintain consistent heat conduction and viscosity for an extended period of time. Particles aggregate and collapse when the zeta potential is low, which lowers the nanofluid’s performance [18]. The zeta potential can be raised and system stability can be guaranteed by adding surfactants. On the other hand, size analysis is a technique that quantifies the size distribution of the particles in the nanofluid. The reliability and effectiveness of the system are directly impacted by the particle size in nanofluids; uniformly sized particles avoid clumping and guarantee consistent fluid properties and heat conduction [19].

Additionally, stability analyses of surfactant-interacted metal-based nanofluids using Density Functional Theory (DFT) and Molecular Dynamics (MD) methods are also available in the literature. The use of GaN and InN nanosheets containing dodecylamine as a surfactant has been investigated as nanofluids to be used in solar plants [20]. Interactions between the sheets and surfactants have been determined using DFT. The optimum interaction point on the surface corresponds to the metallic atom of the sheet and the N atom of the surfactant. Stability analyses of nanofluids based on a eutectic mixture of diphenyl oxide and biphenyl and NiO nanoparticles have been conducted [21]. Two surfactants have been used to analyze the stability of the nanofluids: benzalkonium chloride and 1-octadecanethiol. DFT calculations have been performed to understand surfactant interactions with the fluid and NiO surface. Consistent with the experimental results, nanofluids containing benzalkonium chloride have been favored over those containing 1-octadecanethiol. In another study, the MD method has shown that benzalkonium chloride again has better stabilized NiO nanofluids with respect to 1-octadecanethiol [22]. A MD study based on gold nanofluids in the presence and absence of tetraoctylammonium halide as a surfactant has also been presented [23]. A base fluid consisting of a mixture of biphenyl and diphenyl oxide has been used in the analyses. Theoretical results have indicated that the surfactant plays an active role in improving the thermal properties of the gold nanofluid system by acting as a kind of net around the nanoparticle.

In this study, water-based nanofluids containing Al_2_O_3_ nanoparticles have been prepared using six different surfactants (SDS: sodium dodecyl sulfate, CTAC: cetyltrimethylammonium chloride, PVP: polyvinylpyrrolidone, Tween 80: polyethylene glycol sorbitan monooleate, PVA: polyvinyl alcohol, and Triton X-100: octyl phenol ethoxylate), which represent anionic, cationic, and nonionic characteristics. Zeta potential, particle size analysis, interaction Gibbs free energy, thermal conductivity, dynamic viscosity, and density values have been analyzed together, in a manner different from the existing literature. To the best of our knowledge, no other study has investigated all these parameters in a single nanofluid. Therefore, this study is unique in this respect. Thus, the effect of surfactant type on the colloidal and thermophysical properties of the nanofluid has been systematically analyzed. Additionally, for novelty, nanoparticle–surfactant interactions have been modeled at the molecular level using the DFT method with Gaussian 09 quantum chemical software, and the relationship between the standard interaction Gibbs free energy changes and experimentally measured zeta potentials has been examined.

## 2. Materials and Methods

### 2.1. Nanoparticles and Surfactants

In this study, pure water and Al_2_O_3_ nanoparticles at a 0.5% volume concentration were used as the base fluid. Triton X-100, PVA, Tween 80, CTAC, PVP, and SDS were selected as surfactants. A blank sample without surfactant was prepared to compare results. Table 1 and Table 2 present the properties of the nanoparticles and surfactants obtained from the manufacturer (Sigma-Aldrich, Darmstadt, Germany).

### 2.2. Nanofluid Preparation

**Figure 1:**
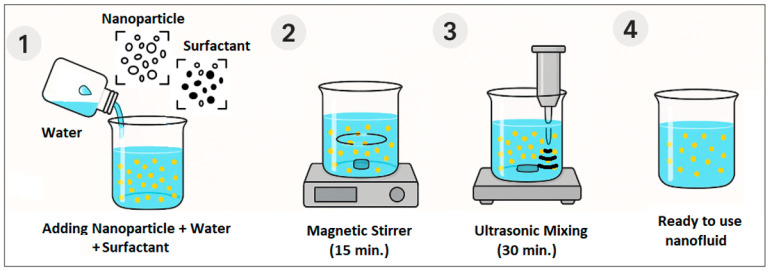
Nanofluid preparation.

**Figure 2:**
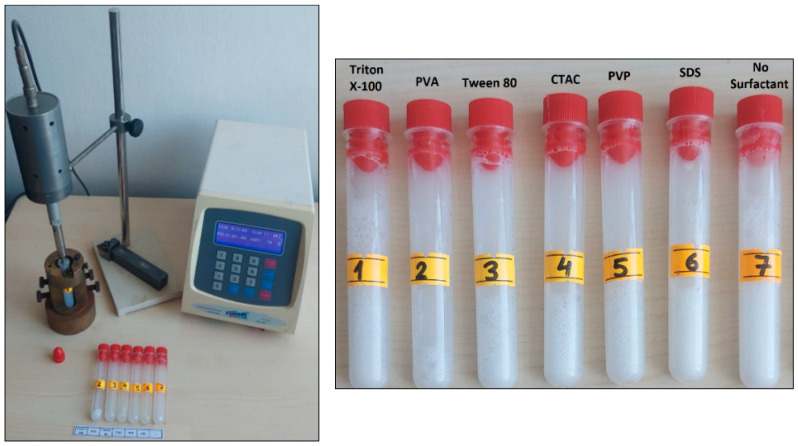
Prepared nanofluids using an ultrasonic homogenizer.

In this study, a water-based, surfactant-added nanofluid was prepared using Al_2_O_3_ nanoparticles at a 0.5% volumetric concentration using a two-step method which is widely employed in the literature. The two-step method is based on the dispersion principle of pre-synthesized nanoparticles in a suitable base fluid and is frequently used in industrial applications [24]. In the first step, Al_2_O_3_ nanoparticles were added to pure water at a 0.5% volumetric concentration, and surfactant was added at 50% of the nanoparticle concentration to reduce the aggregation tendency of the particles. The resulting suspension was stirred for 15 min on a magnetic stirrer (Daihan Wisd MSH-20A, Daejeon, Republic of Korea, 180 × 180 mm plate size, and 1500 rpm maximum stirring speed) to pre-disperse the nanoparticles and ensure effective adsorption of the surfactant onto the particle surface. The samples have then been sonicated for 30 min with 80% vibration amplitude ultrasonic homogenizer (Optic Ivymen System CY-500, Barcelona, Spain, 500 W power, 20 kHz frequency) to break down possible nanoparticle clusters and achieve a more stable, homogeneous, and finer distribution (Figure 1 and Figure 2). Thermal conductivity, dynamic viscosity, density, zeta potential, and particle size measurements were performed to assess the effect of each surfactant used. Then, all were compared with the prepared blank solution. In a previous stability study conducted on Al_2_O_3_ nanofluid with a concentration of 0.5%, it was found that the fluid maintained its stable structure for up to 5 days, and additionally, its thermophysical properties at different temperatures were studied [25,26].

### 2.3. Experimental Measurement of Colloidal Stabilities and Thermophysical Properties

#### 2.3.1. Zeta Potential and Particle Size Analysis

**Figure 3:**
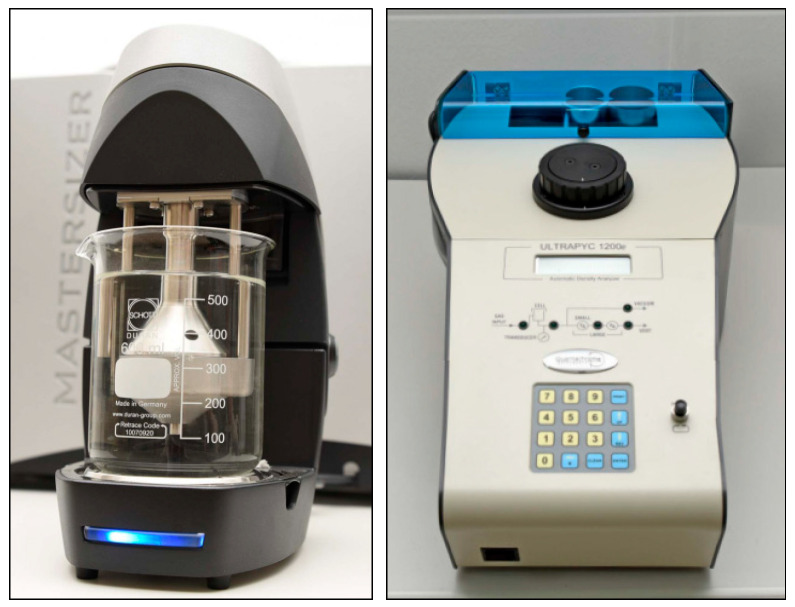
Devices used in the zeta potential (**left**) and particle size analyses (**right**).

In this study, zeta potential and size analyses were performed at Hacettepe University HUNITEK laboratories for seven samples considered (containing blank). Zeta potentials were measured using the Laser Doppler Electrophoresis (LDE) technique with the Zetasizer Nano ZSP device, Malvern, UK (measurement range: 3.8 nm–10 µm) three times, and the average was calculated. Particle sizes were measured using the Dynamic Light Scattering (DLS) technique with the Mastersizer 3000 Device, Malvern, UK (data acquisition speed: 10 kHz, measurement range: 10 nm–3.5 mm, and margin of error: <0.5%) three times, and the average was obtained (Figure 3) [27].

#### 2.3.2. Thermal Conductivity, Dynamic Viscosity, and Density

**Figure 4:**
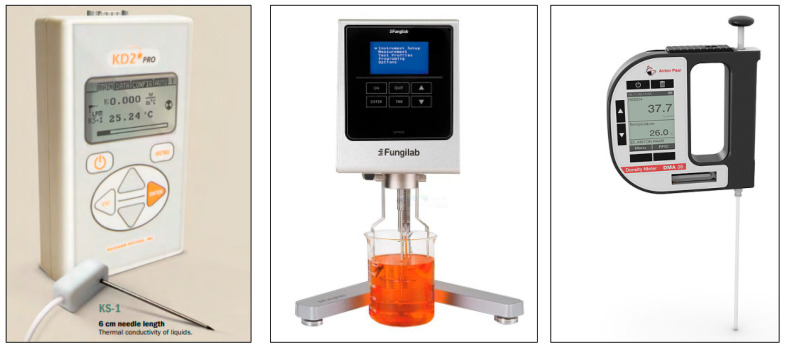
Devices of thermal conductivity (**left**), viscosity (**center**), and density (**right**) used to determine the thermophysical properties of nanofluids.

The thermal conductivity values of the considered seven nanofluids prepared at ambient temperature (approximately 26–27 °C) were measured using the KD2 Decagon Pro Device (accuracy range: ±5% to ±10%, measurement range: 0.02–2.00 W/m·K, and operating temperature range: −50 to 150 °C, with the TR-1 sensor specifically manufactured for liquid samples, diameter: 2.4 mm, length: 100 mm) [28]. The dynamic viscosity measurements of nanofluids were performed at ambient temperature (approximately 25 °C) using the FUNGILAB Smart L Device (measurement accuracy: ±1%, measurement range: 2000–21,333 mPa·s, sample volume: 16 mL, temperature range: −10 to 100 °C, with the LCP/B adapter suitable for low viscosity nanofluids close to water) [29]. The density of the water-based nanofluid was measured using an Anton Paar DMA35 Density Meter (±0.001 g/cm^3^ accuracy, measurement range: 0–2 g/cm^3^, minimum sample volume: 50 mL, temperature range 10–50 °C) [30]. The thermal conductivity, dynamic viscosity, and density values were measured at least three times (Figure 4) and the average values were calculated.

#### 2.3.3. Computational Chemistry Methods

All calculations were carried out in the gas phase and at standard conditions (25 °C, 1 atm) using Gaussian 09 program software [31]. The Gibbs free energy, enthalpy, and entropy changes of the considered Al_2_O_3_–surfactant interactions (∆G°_int_, ∆H°_int_, and ∆S°_int_) were calculated at the DFT B3LYP/LANL2DZ level of theory [32,33]. The thermodynamic calculations included basis set superposition error (BSSE) corrections. BSSE corrections were performed using the Boys and Bernardi counterpoise technique, which arises from the overlapping of the wave functions of the moieties [34]. The linear conformer of Al_2_O_3_ was used throughout the calculations [35]. In this way, the angular bending in Al_2_O_3_ nanoparticles caused by the interaction of the surfactant could be observed easier and practically. Some approaches that provide convenience in the calculations were taken as the basis: polymeric surfactants PVP, PVA, and Triton X-100 monomerically interacted with the Al_2_O_3_ molecule, and the indices of x, y, z, and w were selected as 5 in the Tween 80 molecule for uniformity (x + y + z + w = 20). The energies of all minima considered on the potential energy surface included zero-point energy corrections and existed with no imaginary frequencies, indicating that they are not either transition states or saddle points.

## 3. Results and Discussion

### 3.1. Zeta Potential and Size Analyses of Nanofluids

Table 3 shows the measured zeta potentials and particle sizes of considered Al_2_O_3_–surfactant nanofluids. The case where no surfactant was used is taken as a blank, and percent changes relative to the blank were calculated for the structures. The absolute values of zeta potentials were taken into account throughout the calculations.

**Figure 5:**
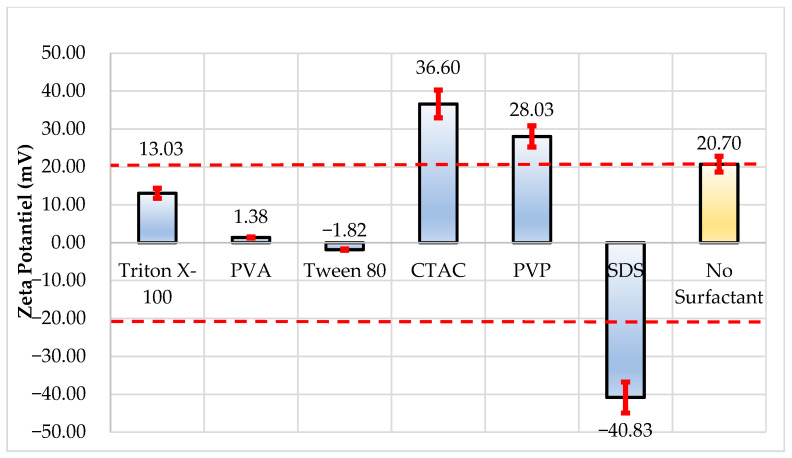
Measured zeta potential values and inaccuracy error bars (±10%).

Zeta potential analyses reveal the electrostatic stability of particles and their potential for interaction with each other. The cationic surfactant CTAC raised the zeta potential by 76.81% compared to the blank (20.70 mV), accomplishing a value of 36.60 mV (Table 3 and Figure 5). This indicates that the electrostatic repulsive forces between the particles have significantly increased, improving colloidal stability. In the same way, the nonionic polymeric surfactant PVP raised the zeta potential to 28.03 mV, which was 35.43% higher than that of the blank. However, the zeta potential was adversely affected by other nonionic surfactants (Triton X-100, PVA, and Tween 80), and notable drops were noted in comparison to the blank (−37.04%, −93.35%, and −91.19%, respectively). This suggests that the surface charge of the particles is neutralized or destabilized by these surfactants. SDS (anionic surfactant), on the other hand, increased the zeta potential to −40.83 mV and represented a 97.26% increase. The negative sign in this case indicates a reversal of the surface charge, signaling the formation of a strong electrostatic repulsion.

**Figure 6:**
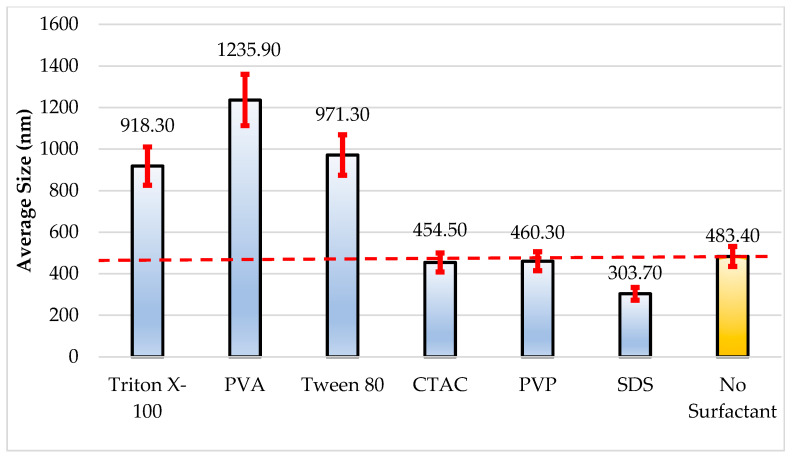
Measured size analysis and inaccuracy error bars (±10%).

When examining the size analysis results in Table 3 and Figure 6, with PVA use, the particle size increased to 1235.90 nm, showing a 155.67% increase with respect to the blank (483.40 nm); which indicates that PVA causes particle aggregation in a certain amount. Similarly, size increases of 89.97% and 100.93% were observed in Triton X-100 (918.30 nm) and Tween 80 (971.30 nm), respectively. In contrast, the use of CTAC (454.50 nm) and PVP (460.30 nm) was observed to decrease the particle size by −5.98% and −4.78%, respectively. In particular, SDS demonstrated a −37.17% decrease, reducing the size to 303.70 nm, which implies that it prevents aggregation by causing particles to repel each other due to its strong negative surface charge.

**Figure 7:**
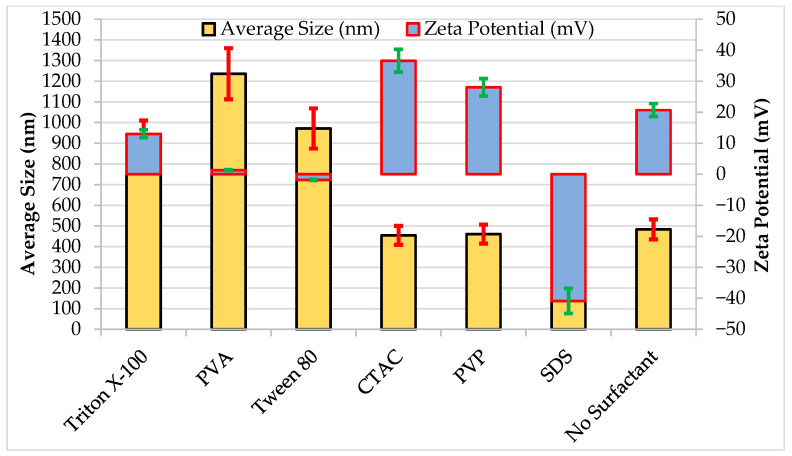
Measured sizes and zeta potentials of the particles and inaccuracy error bars (±10%).

If the zeta potentials and sizes of the particles are examined together as in Figure 7, it can be concluded that CTAC, PVP, and SDS with relatively higher zeta potentials and lower particle sizes are good candidates to prevent aggregation. The other three (Triton X-100, PVA, and Tween 80), on the other hand, with relatively lower zeta potentials and higher particle sizes, can be classified as disadvantageous surfactants.

### 3.2. Thermophysical Properties of Nanofluids

The thermophysical behavior of nanofluids is determined by fundamental fluid properties; primarily thermal conductivity, dynamic viscosity, and density. Thermal conductivity is the most critical parameter directly affecting the heat transfer performance of the nanofluid, and it increases with the addition of nanoparticles compared to the base fluid. Dynamic viscosity determines the flow resistance and consequently the pressure drop and pumping power requirement; therefore, it needs to be evaluated together with the increase in thermal conductivity. Density, on the other hand, is decisive in the flow regime and inertial effects, and plays an important role in Reynolds number and mass flow rate calculations. The combined analysis of these parameters enables accurate evaluation of the actual performance of nanofluids in heat transfer applications. In this context, experimental measurements were carried out for seven different nanofluids to quantitatively determine these thermophysical parameters.

Table 4 shows the measured thermal conductivity values of the prepared nanofluids and the changes in percent compared to the blank. In particular, the use of SDS, CTAC, and PVP (with 9.36%, 5.47%, and 3.53% relative values) increased the thermal conductivity a significant amount. However, Triton X-100 and Tween 80 (with 1.64% and 1.33% relative values, respectively) slightly increased the thermal conductivity, while a moderate decrease was observed with the use of PVA (−2.25%).

Table 5 presents the measured dynamic viscosity values of nanofluids along with the changes in percent compared to the blank. The use of surfactant increased the dynamic viscosities of all the considered nanofluids up to 31.28% (in the order of SDS (10.55%) < CTAC (11.11%) < Triton X-100 (14.11%) < Tween 80 (21.35%) < PVA (24.56%) < PVP (31.28%). This expected increase arises from the stronger molecular interactions formed by the help of the surfactants and thereby increasing the fluid’s resistance capacity. The results demonstrate that the use of more stable and lower viscosity SDS and CTAC surfactants may play a critical role in the improvement of fluidity and pumping performance of nanofluids. The measured density values are given in Table 6. According to these values, the density was minimally affected by surfactant addition. In general, no significant change was observed in case of surfactant use.

**Figure 8:**
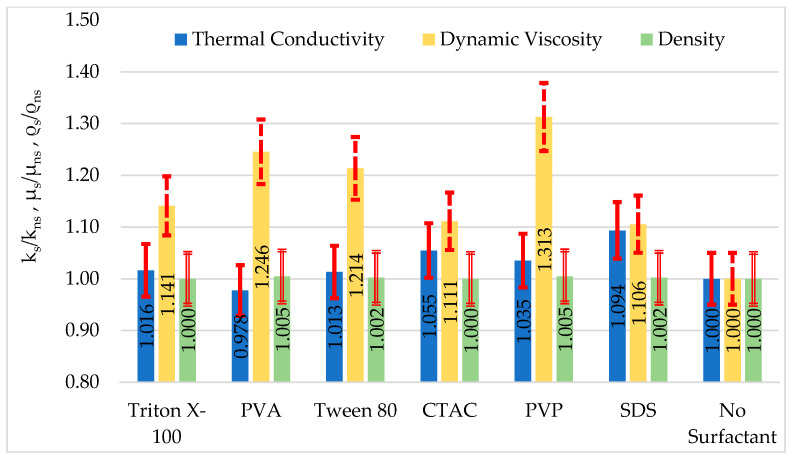
Relative ratios of thermal conductivity, dynamic viscosity, and density values compared to the blank (s: with surfactant, ns: no surfactant) and inaccuracy error bars (±10%).

Figure 8 shows the relative ratios of thermal conductivity (k_s_/k_ns_), dynamic viscosity (μ_s_/μ_ns_), and density values (ρ_s_/ρ_ns_) of the structures all together compared to the blank. When Figure 7 and Figure 8 are examined together, it can be concluded that Al_2_O_3_-SDS and Al_2_O_3_-CTAC nanofluids can be used alternatively in cooling systems due to their high colloidal stability, high thermal conductivity, and low viscosity. Figure 8 shows a comparative visualization of the thermophysical properties of nanofluids prepared with a base fluid without surfactants and those prepared using different surfactants. The main reason for not starting the vertical axis from zero was to allow for a clearer and more distinguishable representation of the percent changes and small differences between the fluids under study. Accordingly, the axis range was limited to 0.8–1.5 to better represent the distribution range of the measured values. Additionally, when the obtained thermophysical values of the prepared nanofluids were compared with those in the literature, it was concluded that the rates of increase in thermal conductivity and viscosity were similar to those in past studies [36,37].

#### Uncertainty Analysis

In this study, the thermal conductivity, dynamic viscosity, and density values of Al_2_O_3_ nanofluids containing different surfactants were measured using KD2 Pro (Decagon Devices), FUNGILAB Smart L, and Anton Paar DMA 35 instruments, respectively. Measurement uncertainties were evaluated based on the accuracy values given by the instrument manufacturers and the experimental results. The total uncertainty of a measured quantity were calculated using the sum of roots method as shown in Equation (1).

(1) $$\frac{u_{R}}{R}=\sqrt{\sum_{i=1}^{n}{\left(\frac{U_{x_{i}}}{x_{i}}\right)}^{2}}$$

In Equation (1), *R* represents the measured physical quantity, *U_R_* represents the total uncertainty of this quantity, *x_i_* represents the independent measurement parameters, $U_{x_{i}}$ represents the uncertainty of each parameter, and *n* represents the number of independent parameters.

In line with this approach, the average thermal conductivity value in Table 7 was 0.670 W/m·K, and considering the ±5% accuracy of the KD2 Pro device, the uncertainty was given as ±0.034 W/m·K. The average viscosity value measured with the FUNGILAB Smart L device was 1.11 mPa·s, and considering the ±1% accuracy, the uncertainty was determined as ±0.01 mPa·s. In density measurements, the ±0.001 g/cm^3^ accuracy of the Anton Paar DMA 35 device was considered, and the uncertainty was calculated as ±1 kg/m^3^ for an average density value of 1007.38 kg/m^3^.

The physical significance of the measurements was evaluated by comparing the observed maximum differences for different surfactants with their respective measurement uncertainties. The observed difference of 0.076 W/m·K in thermal conductivity was above the uncertainty of ±0.034 W/m·K. For viscosity, the maximum difference was 0.299 mPa·s, significantly exceeding the uncertainty of ±0.01 mPa·s. Similarly, the observed difference of 4.67 kg/m^3^ in density was above the uncertainty of ±1 kg/m^3^. These results have demonstrated that the experimentally obtained differences were not due to measurement errors and that surfactants have a physically significant effect on the thermophysical properties of nanofluids.

### 3.3. Computational Chemistry Results

#### 3.3.1. Structure Optimizations of Al_2_O_3_–Surfactant Interactions

**Figure 9:**
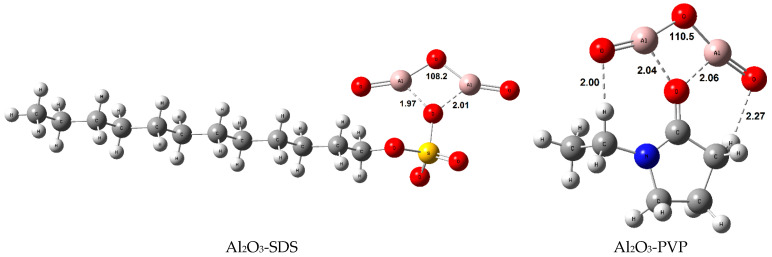

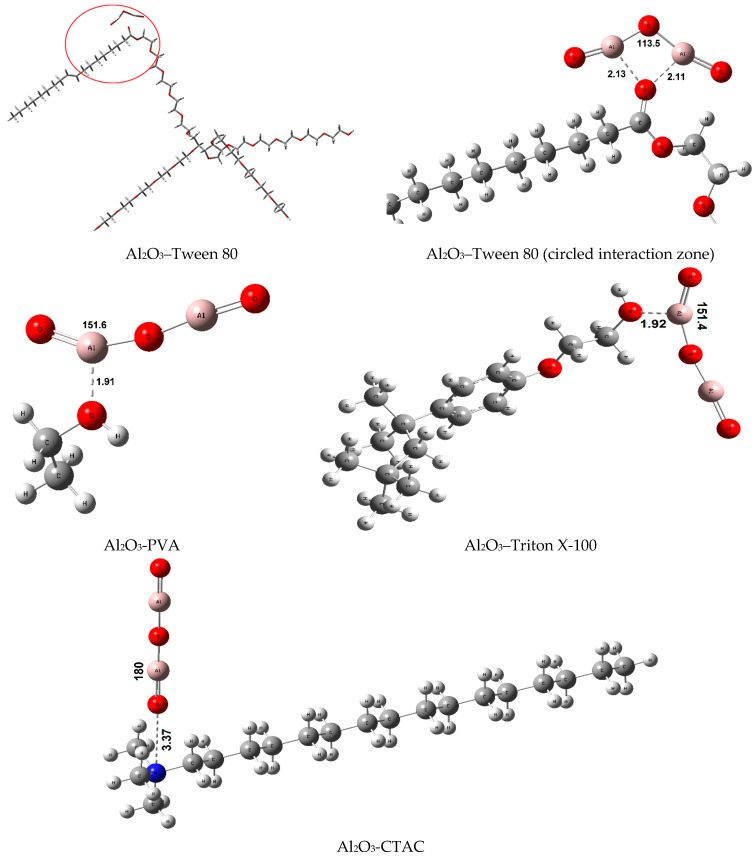
The molecularly modeled and optimized Al_2_O_3_–surfactant nanofluids in decreasing thermodynamic stability order (bond lengths in Å and Al_2_O_3_ bending angles in degrees).

The molecularly modeled and optimized Al_2_O_3_–surfactant nanofluids are presented in Figure 9. Al_2_O_3_ interacted tetragonally with the surfactants SDS, PVP, and Tween 80, respectively. Furthermore, these interactions have disrupted the linear structure of Al_2_O_3_ and formed significant angular bending. Among these three surfactants, the shortest interaction distance and the highest angle bending were observed in SDS, PVP, and Tween 80, respectively. From these observations, it can be concluded that structural interaction also increased in the same order. Conversely, a one-side interaction was observed in the Al_2_O_3_-PVA and Al_2_O_3_–Triton X-100 structures. The interaction distances were slightly closer than in the first group, but the angular bending in Al_2_O_3_ was significantly weaker in both. Therefore, it implies that PVA and Triton X-100 interact much more weakly with Al_2_O_3_ compared to the first three surfactants. Finally, the weakest interaction was observed in the Al_2_O_3_-CTAC structure due to the relatively long interaction distance. As a result, the Al_2_O_3_ molecule maintained its linearity, and no angular bending occurred. In summary, the guessed thermodynamic stability order obtained from the Al_2_O_3_–surfactant optimizations (geometries) was Al_2_O_3_-SDS > Al_2_O_3_-PVP > Al_2_O_3_–Tween 80 > Al_2_O_3_-PVA > Al_2_O_3_–Triton X-100 > Al_2_O_3_-CTAC. In the next section, the thermodynamic stabilities are discussed computationally in terms of Gibbs free energy of interaction (∆G°_int_), interaction enthalpy (∆H°_int_), and interaction entropy (∆S°_int_) at standard conditions to approve the predictions.

#### 3.3.2. Thermodynamic Calculations of Al_2_O_3_-Surfactant Interactions

The standard Gibbs free energy of interaction (ΔG°_int_) between a metal-based nanoparticle and a surfactant is an important parameter for colloidal stability, and its calculation, in addition to the zeta potential, can be quite useful in understanding the behavior of the system. The tendency of a surfactant to interact with a metal-based nanoparticle is expressed thermodynamically as ΔG°. If this value is negative (ΔG° < 0), the interaction is spontaneous. A more negative ΔG° value indicates a stronger interaction and better surface coverage by the surfactant, which in turn increases steric stability. In this context, the strong and stable interaction of the surfactant with the surface not only indirectly affects the zeta potential by affecting the surface charge distribution but also physically impedes particle convergence by creating steric hindrance. In addition, while the zeta potential provides information about the electrostatic stability of the colloid, the ΔG° value provides additional information about its long-term stability. Therefore, calculating ΔG° is quite useful for a more in-depth understanding of colloidal stability. Among the structures examined, the most stable interaction was observed in the Al_2_O_3_-SDS structure thermodynamically, with an energy change of approximately −400 kJ/mol (Table 8). The second-strongest interaction group included the Al_2_O_3_-PVP and Al_2_O_3_–Tween 80 structures. The energy change at the end of the interactions decreased by almost half compared to Al_2_O_3_-SDS, reaching approximately −200 kJ/mol for both. The third group consisted of the Al_2_O_3_-PVA and Al_2_O_3_–Triton X-100 structures in terms of stability. The energy change for both was approximately −100 kJ/mol, almost one-fourth of that for Al_2_O_3_-SDS. Thermodynamically, the most unstable interaction belonged to the Al_2_O_3_-CTAC structure, and the interaction energy change dropped to ~−50 kJ/mol. This was approximately one-eighth of the energy released by the Al_2_O_3_-SDS interaction and was quite small compared to the others. As a result, the most stable interaction was obtained with the anionic surfactant SDS, while the least stable interaction was obtained with the cationic surfactant CTAC. On the other hand, nonionic surfactants demonstrated moderately stable interactions.

The stability order of the Al_2_O_3_–surfactant structures in terms of standard interaction enthalpies (ΔH°_int_) was the same as in standard interaction Gibbs free energies (ΔG°_int_) (Table 8). It was noteworthy that the ΔH°_int_ values were more negative than the ΔG°_int_ values, indicating that the energy difference was spent on the entropic rearrangement of the Al_2_O_3_–surfactant interactions (ΔG°_int_ = ΔH°_int_ − T·ΔS°_int_). The interaction entropy (ΔS°_int_) was negative for all the considered structures, as the Al_2_O_3_ and surfactants became more ordered upon chemical interactions. The most ordered structure was Al_2_O_3_-PVP, while the least ordered structure was Al_2_O_3_–Tween 80. The hydrogen bonds are thought to be effective in ordered Al_2_O_3_-PVP structure. However, the branched and bulky structure of Tween 80 in Al_2_O_3_–Tween 80 interaction diminishes ordering due to strong steric effects (Figure 9). Furthermore, although the calculations in the study have not considered solvent and solvent-dependent dispersion effects, and relatively simple chemical interaction models were designed and used, it is clear that gas-phase calculations performed in vacuum can be illuminating about intermolecular interactions and their thermodynamic trends. Undoubtedly, the chemicals will be much more stable in a solvent environment, but the ordering tendencies of ∆G°_int_, ∆H°_int_, and ∆S°_int_ will be similar.

As discussed in previous sections, it is quite normal for the zeta potential order to differ from the thermodynamic stability order for the colloids in question. This is because these two metrics reflect different physicochemical properties. Zeta potential indicates the surface charge of particles in suspension, as well as the impact of this charge on the surrounding liquid. In other words, it only measures electrostatic stability. Generally, a higher absolute zeta potential means stronger electrostatic repulsion, less aggregation, and better colloidal stability. Thermodynamic stability, on the other hand, indicates the energetic stability of the system. The lower a system’s Gibbs free energy, the more stable it is. This is a much more comprehensive measure, including not only electrostatic effects but also factors such as hydrogen bonds, van der Waals forces, steric effects, and hydrophobic interactions. Therefore, a nanofluid may have a high zeta potential, but if steric effects are weak, it may become thermodynamically unstable in a long term. For these reasons, surfactants that rank higher in both orders (zeta potential and ΔG°_int_) are prime candidates.

The absolute zeta potential order presented in the previous sections is as follows: Al_2_O_3_-SDS (~41 mV) > Al_2_O_3_-CTAC (~37 mV) > Al_2_O_3_-PVP (~28 mV) > Al_2_O_3_–Triton X-100 (~13 mV) > Al_2_O_3_–Tween 80 (~1.8 mV) > Al_2_O_3_-PVA (~1.4 mV). Based solely on zeta potential, colloids with higher values with respect to the blank (~21 mV) are considered advantageous (SDS, CTAC, and PVP). However, at this point, the calculated interaction Gibbs free energies also need to be considered. Accordingly, the energy changes are in the order of Al_2_O_3_-SDS (~−399 kJ/mol) > Al_2_O_3_-PVP (~−225 kJ/mol) > Al_2_O_3_–Tween 80 (~−213 kJ/mol) > Al_2_O_3_-PVA (~−117 kJ/mol) > Al_2_O_3_–Triton X-100 (~−101 kJ/mol) > Al_2_O_3_-CTAC (~−48 kJ/mol). SDS and PVP, which are at the top of both orders, are the most ideal surfactants for the experimental conditions studied. Although CTAC exhibited a high zeta potential, it had quite low thermodynamic stability. The opposite was observed for the Tween 80 molecule. Its thermodynamic stability was high; however, its zeta potential was very low. Furthermore, despite having moderate thermodynamic stability, PVA and Triton X-100 exhibited low zeta potentials. Therefore, CTAC, Tween 80, PVA, and Triton X-100 may not be considered suitable candidates for interacting with Al_2_O_3_.

The experimentally and theoretically determined colloidal stabilities and measured thermophysical properties performed up to now are summarized in Table 9. In the table, the most advantageous surfactants with respect to the blank are recommended for each of the parameters considered. As higher zeta potential, higher thermal conductivity, and lower particle size are critical experimental parameters for nanofluids, SDS > CTAC > PVP order predominates. The order changes to PVP > CTAC > SDS when higher dynamic viscosity is taken into account, i.e., in machining applications. Additionally, all the interactions mentioned with SDS, CTAC, and PVP occur spontaneously according to computational analyses. The order of stability of the surfactants is SDS > PVP > CTAC, with decreasing negative ΔG°_int_ values.

## 4. Conclusions

The findings show that choosing the right surfactant is essential for creating nanofluids as it affects both the zeta potential and particle size, which in turn can have an impact on long-term stability, and macroscopic characteristics like thermal conductivity and dynamic viscosity. Accordingly, SDS, CTAC, and PVP are recommended, respectively, for colloidal stability (higher zeta potential) and less aggregation (lower size). The same order is also maintained for thermophysical properties (higher thermal conductivity and lower dynamic viscosity). Note that higher thermal conductivity is an important parameter for heating–cooling systems whereas lower dynamic viscosity is more practical in fluid pumping applications. However, since the increase in dynamic viscosity is significantly more pronounced in PVP-containing nanofluids, the use of PVP-based nanofluids is considered more suitable and is therefore recommended for machining processes (e.g., CNC applications). In addition, SDS, CTAC, and PVP have negative interaction Gibbs free energies which imply that they all interact simultaneously with Al_2_O_3_. Thermodynamically more stable SDS and PVP can be used if long-term stability is important; on the other hand, that of less stable CTAC can be used in short-term applications. In general, this study demonstrates that surfactant selection plays a critical role depending on what it will be used for (heat transfer applications, industrial applications, electronics and micro-systems, automotive and aerospace sectors, materials and nanotechnology fields, etc.).

## Figures and Tables

**Table 1:** Properties of the nanoparticles.

Nanoparticle Type	Density (g/mL)	Particle Size (nm)	Purity (%)	Shape	Color
Aluminum oxide (Al_2_O_3_)	3.89	13	99.5	Nearly spherical	White

**Table 2:** Properties of the surfactants.

Surfactant	Chemical Name	Type	Purity (%)	Density (g/mL)	Charge	Solubility in Water
Triton X-100	octyl phenol ethoxylate	nonionic	≥99	1.07	neutral	high
PVA	polyvinyl alcohol	nonionic	≥98	1.25	neutral	high
Tween 80	polyethylene glycol sorbitan monooleate	nonionic	≥95	1.06	neutral	high
CTAC	cetyltrimethylammonium chloride	cationic	≥98	0.90	+	medium
PVP	polyvinylpyrrolidone	nonionic	≥99	1.22	neutral	high
SDS	sodium dodecyl sulfate	anionic	≥99	1.01	−	high

**Table 3:** Zeta potential and size analyses results.

Used Surfactant	Average Zeta Potential (mV)	Percent Change Relative to Blank (%)	Size Analysis (nm)	Percent Change Relative to Blank (%)
Triton X-100	13.03	−37.04	918.30	89.97
PVA	1.38	−93.35	1235.90	155.67
Tween 80	−1.82	−91.19	971.30	100.93
CTAC	36.60	76.81	454.50	−5.98
PVP	28.03	35.43	460.30	−4.78
SDS	−40.83	97.26	303.70	−37.17
No Surfactant	20.70	-	483.40	-

**Table 4:** Measured thermal conductivity values.

Surfactant	Temperature (°C)	Thermal Conductivity (W/m·K)	Percent Change Relative to Blank (%)
Triton X-100	26.71	0.663	1.64
PVA	26.21	0.637	−2.25
Tween 80	26.05	0.661	1.33
CTAC	26.39	0.688	5.47
PVP	26.18	0.675	3.53
SDS	26.89	0.713	9.36
No Surfactant	26.18	0.652	-

**Table 5:** Measured dynamic viscosity values.

Surfactant	Temperature (°C)	Dynamic Viscosity (mPa·s)	Percent Change Relative to Blank (%)
Triton X-100	24.53	1.092	14.11
PVA	24.81	1.192	24.56
Tween 80	25.03	1.161	21.35
CTAC	25.03	1.063	11.11
PVP	24.26	1.256	31.28
SDS	24.90	1.058	10.55
No Surfactant	24.35	0.957	-

**Table 6:** Measured density values.

Surfactant	Temperature (°C)	Density (kg/m^3^)	Percent Change Relative to Blank (%)
Triton X-100	25.97	1005.50	0.01
PVA	25.73	1010.03	0.46
Tween 80	25.53	1007.53	0.21
CTAC	24.93	1005.43	0.00
PVP	25.57	1010.17	0.47
SDS	25.13	1007.60	0.22
No Surfactant	25.93	1005.40	-

**Table 7:** Uncertainty analysis and physical significance of thermophysical properties.

Property	Average Value	Absolute Uncertainty	Maximum Observed Difference	Physical Significance
Thermal conductivity k (W/m·K)	0.670 W/m·K	±0.034 W/m·K	0.076 W/m·K	Difference > uncertainty (significant)
Dynamic viscosity, μ (mPa·s)	1.11 mPa·s	±0.01 mPa·s	0.299 mPa·s	Difference > uncertainty (highly significant)
Density, ρ (kg/m^3^)	1007.38 kg/m^3^	±1 kg/m^3^	4.67 kg/m^3^	Difference > uncertainty (significant)

**Table 8:** Thermodynamic results of Al_2_O_3_–surfactant interactions at the DFT B3LYP/LANL2DZ theoretical level.

Structure	∆H°_int_ (kJ·mol^−1^)	∆S°_int_ (kJ·mol^−1^·K^−1^)	∆S°_int_ (J·mol^−1^·K^−1^)	∆G°_int_ (kJ·mol^−1^)
Al_2_O_3_-SDS	−440.909	−0.140	−140	−399.027
Al_2_O_3_-PVP	−284.408	−0.201	−201	−224.570
Al_2_O_3_–Tween 80	−224.611	−0.039	−39	−212.970
Al_2_O_3_-PVA	−160.159	−0.146	−146	−116.531
Al_2_O_3_–Triton X-100	−140.722	−0.132	−132	−101.290
Al_2_O_3_-CTAC	−66.407	−0.063	−63	−47.680

**Table 9:** Recommended most advantageous surfactants with respect to the blank.

Surfactant	Absolute Increase in Zeta Potential (%)	Absolute Decrease in Size (%)	Thermal Conductivity Increase (%)	Dynamic Viscosity Increase (%)	Interaction Gibbs Free Energy (kJ/mol)
SDS	97.26	37.17	9.36	10.55	−399.03
CTAC	76.81	5.98	5.47	11.11	−47.68
PVP	35.43	4.78	3.53	31.28	−224.57

## Data Availability

The original contributions presented in this study are included in the article. Further inquiries can be directed to the corresponding author.
